# Targeting DLL3: Innovative Strategies for Tumor Treatment

**DOI:** 10.3390/pharmaceutics17040520

**Published:** 2025-04-16

**Authors:** Hui Wang, Tong Zheng, Dan Xu, Chao Sun, Daqing Huang, Xiongxiong Liu

**Affiliations:** 1 Institute of Modern Physics, Chinese Academy of Sciences, Lanzhou 730000, China; wangh08@impcas.ac.cn (H.W.); zhengtong24@mails.ucas.ac.cn (T.Z.); xudan@impcas.ac.cn (D.X.); sunchao@impcas.ac.cn (C.S.); 2 Key Laboratory of Heavy Ion Radiation Biology and Medicine, Chinese Academy of Sciences, Lanzhou 730000, China; 3 Key Laboratory of Basic Research on Heavy Ion Radiation Application in Medicine, Lanzhou 730000, China; 4 University of Chinese Academy of Sciences, Beijing 100049, China; 5 School of Nuclear Science and Engineering, East China University of Technology, Nanchang 330013, China; 13629502707@163.com

**Keywords:** DLL3, targeted therapy, solid tumor, clinical trials

## Abstract

Delta-like 3 (DLL3) is an oncogenic protein aberrantly expressed in several tumors, particularly in small-cell lung cancer. DLL3-targeted therapies have recently made significant progress, demonstrating promising preclinical and clinical efficacy. This review aims to explore the mechanisms, challenges, and future opportunities associated with therapies targeting DLL3 for cancer treatment. The biological characteristics of DLL3 and its role in the Notch signaling pathway are introduced first, delving into the role of DLL3 in tumorigenesis and cancer progression. Next, current therapeutic approaches targeting DLL3 are described, including antibody–drug conjugates, T cell engagers, chimeric antigen receptor T cells, and radiopharmaceutical therapy, highlighting their effectiveness and safety in clinical trials. Despite the promising prospects, difficulties remain in the use of DLL3 as a therapeutic target due to tumor heterogeneity, the development of resistance, potential adverse effects, and barriers to patient stratification. Therefore, the potential of combination therapies, the use of innovative drug delivery systems, and ongoing clinical trial advancements are also discussed. Finally, the potential of DLL3-targeted therapies is summarized, highlighting the importance of multidisciplinary research to guide the clinical application and optimization of this emerging treatment strategy. These approaches might provide new therapeutic options, potentially starting a new era in cancer treatment.

## 1. Introduction

Cancer represents a major, significant challenge in the 21st century, with implications for society, public health, and economy. Nearly 20 million new cancer cases were reported globally in 2022, accompanied by an estimated 9.7 million deaths from this disease [1]. Traditional treatment methods, such as surgery, chemotherapy, and radiotherapy, have been the mainstays of cancer management for decades [2]. However, these approaches often cause significant side effects and variable efficacy in different patients and tumor types. These limitations have led to a shift towards precision medicine, which is based on the personalized treatments based on the characteristics of each patient and their tumor [3,4]. Recent advances in the understanding of cancer biology have led to the development of therapies that specifically target cancer-associated molecules to improve treatment efficacy while minimizing the damage to normal cells by inhibiting specific signaling pathways required for tumor survival and growth [5,6,7].

Delta-like canonical Notch ligand 3 (DLL3) is a member of the Notch ligand family involved in cell signaling. It is typically expressed in neural and neuroendocrine tissues during embryonic development, with no or low expression in healthy adult tissues, while it is present on the surface of tumor cells [8,9]. This characteristic makes DLL3 an attractive target for cancer therapy. DLL3 overexpression confers significant survival advantages to tumor cells since it facilitates cell proliferation and metastasis [10]. There is considerable interest in the development of targeted therapies against DLL3, including antibody–drug conjugates (ADCs) and engineered T cell therapies (e.g., chimeric antigen receptor T), both relying on the selective expression of DLL3 in cancer cells, allowing the targeted delivery of therapeutic agents directly to the tumor.

This review aims to summarize the biological role of DLL3, its expression patterns, signaling mechanisms, and its potential role as a therapeutic target in cancer treatment, concluding with an outlook on DLL3-targeted therapies for the treatment of tumors that express it.

## 2. Biological Characteristics of DLL3

### 2.1. Structure and Expression of DLL3

**Figure 1:**
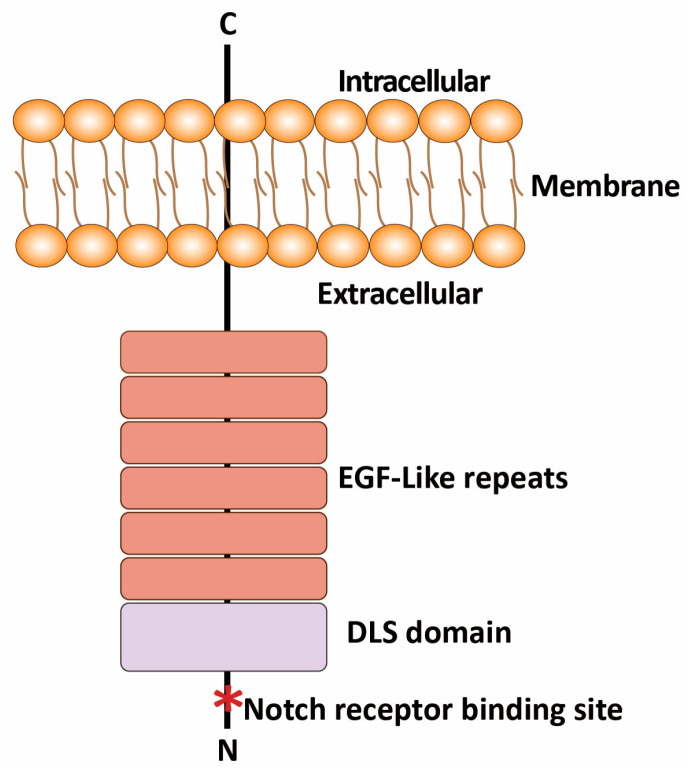
A schematic representation of the DLL3 structure. The domains of DLL3, including the extracellular EGF-like repeats, the Delta/Serrate/LAG-2 (DLS) domain which is critical for ligand–receptor interactions, and the N-terminus (N) and C-terminus (C), are labeled, highlighting that DLL3 is a single transmembrane protein.

DLL3 is a single transmembrane protein consisting of 619 amino acids. Its complete structure includes an extracellular Delta/Serrate/Lag2 (DSL) domain and six epidermal growth factor-like (EGF-like) repeats, as well as a short cytoplasmic domain [11]. The DSL at the N-terminus of the extracellular domain is highly conserved and is crucial for Notch receptor binding (Figure 1). Unlike DLL1 and DLL4, DLL3 lacks Notch receptor activation capability and demonstrates endogenous Notch signaling inhibition and prevents Notch receptor activity [12].

**Figure 2:**
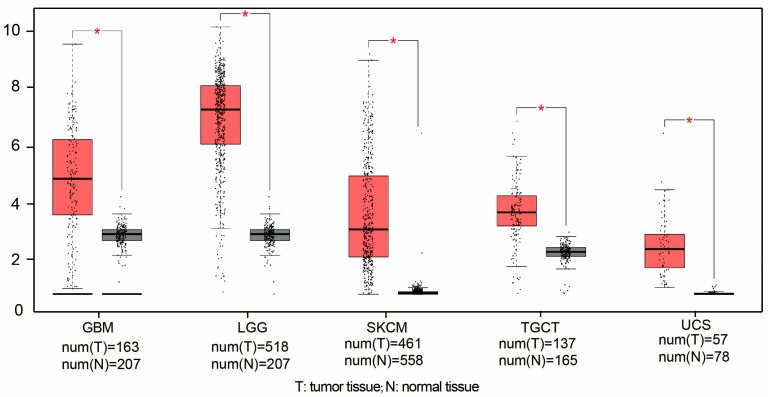
DLL3 expression in tumor and adjacent normal tissues (data source: TCGA and GTEx). GBM: glioblastoma; LGG: lower-grade glioma; SKCM: skin cutaneous melanoma; TGCT: tenosynovial giant-cell tumor; UCS: uterine carcinosarcoma. * *p* < 0.05.

DLL3 plays an important regulatory role in embryonic development during pregnancy, which regulates neural tube closure through the suppression of the Notch signaling pathway. DLL3 gene mutations in mouse models result in the characteristic “pudgy” phenotype, manifested as severe developmental abnormalities of the spine and ribs [13]. Indeed, DLL3 under normal physiological conditions effectively inhibits the activation of the Notch pathway through its interaction with Notch receptors, simultaneously guiding or retaining these activators in late endosomal/lysosomal regions or in the Golgi apparatus, ultimately preventing their appearance on the cell surface. However, DLL3 is selectively expressed in the cell surface of specific tumors, especially in small-cell lung cancer (SCLC) and neuroendocrine tumors. DLL3 is expressed in approximately 80% of patients with primary and metastatic SCLC, making it a potential therapeutic target [14,15,16,17]. In addition to SCLC, DLL3 is overexpressed in other tumor cells, such as stomach cancer cells [18], isocitrate dehydrogenase-mutant glioma [19], gastrointestinal neuroendocrine cancer [20], small-cell bladder cancer [21], and some types of breast cancer [22]. The Gene Expression Profiling Interactive Analysis (GEPIA) 2.0 data platform (http://gepia2.cancer-pku.cn/) was used to analyze the expression of DLL3 in other tumors. DLL3 expression is significantly higher in glioblastoma, lower-grade glioma, skin cutaneous melanoma, tenosynovial giant-cell tumors, and uterine carcinosarcoma than in normal tissues, as shown in Figure 2. Moreover, DLL3 expression is correlated with aggressive disease, suggesting its involvement in determining a more malignant phenotype [23,24]. DLL3 is also present in circulating tumor cells and in blood samples from cancer patients [25,26,27,28]. For these reasons, DLL3 is being explored as a biomarker for the diagnosis and prognosis of several tumors, as well as a target for new therapeutic strategies [25,29]. Its reduced expression in normal tissues compared to cancerous ones makes it an attractive target for therapies aimed at minimizing damage to healthy cells.

### 2.2. Role of DLL3 in Notch Signaling Pathway

The Notch signaling pathway is highly conserved and involved in malignant transformation and cell proliferation and used as a biomarker for cancer progression and prognosis [30,31,32]. The pathway starts with the binding of one of the five ligands (Jagged 1 [Jag 1], Jag 2, DLL1, DLL3, and DLL4) with one of the four receptors (Notch 1–4) [33,34]. The interaction with Notch receptors leads to the inhibition of Notch activation, preventing the downstream signaling events that influence cell differentiation [35]. The aberrant high DLL3 expression in SCLC leads to its binding to Notch receptors and the disruption of the balance of Notch signaling, resulting in the downregulation of the Notch target genes HES1 and HEY1, promoting the development of SCLC [36]. The activation of Notch 1 increases E-cadherin expression, consequently increasing cell adhesion and subsequently suppressing the expression of the EMT-related genes snail, slug, twist, and vimentin, ultimately decreasing cell motility and invasion [37]. Thus, DLL3 promotes migration and invasion in SCLC by modulating Notch 1 [10]. The binding of DLL3 to Notch receptors is an event also observed in other tumors. For instance, DLL3/Notch 2 are involved in the regulation of proliferation and invasion of pituitary adenomas [38], DLL3/Notch 2/Notch 4 in the survival and growth of melanoma cells [39], and DLL3/Notch 2/Notch 3 in the proliferation and differentiation of ovarian cancer cells, being associated with poor survival [40]. Therefore, DLL3 binds to various Notch receptors, exerting multiple roles including cell proliferation, differentiation, and apoptosis.

## 3. DLL3 Mechanism of Action Inducing Cancer Progression and Treatment

### 3.1. DLL3 in Tumorigenesis and Progression

DLL3 is involved in tumorigenesis, particularly in SCLC and large-cell neuroendocrine cancer where it is overexpressed [23,41]. DLL3 inhibits Notch signaling in neighboring cells, creating a microenvironment favorable to tumor growth and progression [10,42]. DLL3 promotes SCLC proliferation through the activation of alternative signaling pathways while suppressing apoptosis. Moreover, DLL3 expression can be a potential new tumor marker for the early diagnosis of endometrial cancer and an independent predictor factor of poor survival in these patients [43]. Furthermore, DLL3 may promote tumor progression by affecting the infiltration of B cells, neutrophils, and T cells in the tumor microenvironment [22,44]. Targeting DLL3 may be a promising therapeutic strategy against the progression of inflammation-exacerbated melanoma by decreasing the expression of MMP1 and MMP9, vascular endothelial growth factor, tumor necrosis factor (TNF)-α, and interleukin (IL)-6 [39,45]. DLL3 modulates cell–cell interaction and may interact with various extracellular matrix (ECM) components, further promoting an invasive phenotype.

Epithelial-to-mesenchymal transition (EMT) induces cancer metastasis, since epithelial cells lose their cell–cell adhesion properties and gain migratory and invasive characteristics [46]. DLL3 expression in preclinical models promotes SCLC migration and invasion through a mechanism that involves the control of the EMT protein snail [10], while DLL3 silencing reverses the EMT process in SCLC cells [47]. DLL3 knockdown inhibits Twist1 expression, a factor involved in the regulation of EMT, as well as the EMT hallmarks slug, N-cadherin, and vimentin, reducing the migration and invasion of melanoma cells [39]. DLL3 inhibits the activation of Notch 1, thus reversing the EMT process and inhibiting the migration and invasion of breast cancer cells by inducing slug expression [48,49]. In vitro studies suggest that DLL3 expression is reduced in glioma cells, and the restoration of its expression inhibits the survival, proliferation, and invasion of these cells [50,51]. DLL3 expression in medullary thyroid carcinoma is a reliable surrogate marker for stromal desmoplasia and lymph node metastasis and may be an indicator of an aggressive clinical behavior [52]. The aforementioned activation leads to the transcriptional upregulation of genes that facilitate EMT, allowing the detachment of cells from the primary tumor that invade the surrounding tissues, ultimately leading to metastasis. Indeed, DLL3 overexpression in SCLC is linked with an increased metastatic potential and distant organ involvement in SCLC.

### 3.2. Interaction of DLL3 with Other Oncogenic Signaling Pathways

**Figure 3:**
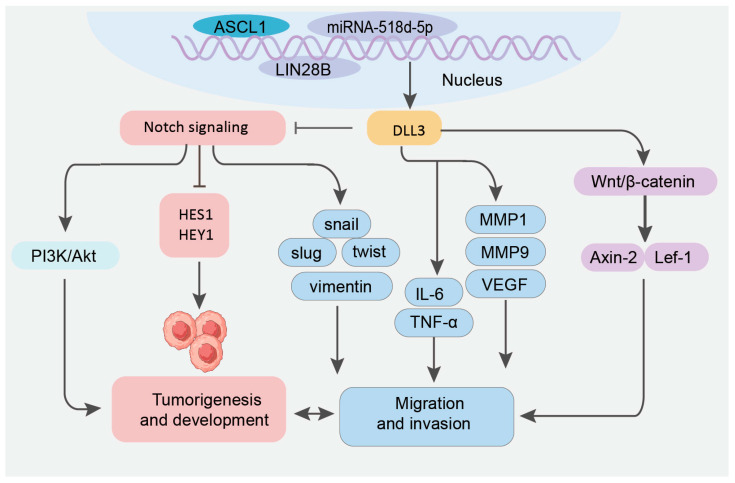
The regulatory network of DLL3 in cellular signaling. DLL3 involves key molecular interactions, including its modulation by ASCL1 and miRNA-518d-5p and cross-talk with Notch, PI3K/Akt, and Wnt/β-catenin pathways. DLL3-mediated Notch inhibition coupled with Wnt activation (through Wnt-1/4, Axin-2, and Lef-1 upregulation) and PI3K/Akt stimulation drives tumor progression by inducing EMT markers (snail, twist, vimentin), matrix metalloproteinases (MMP1 and MMP9), and pro-angiogenic factors (VEGF), while promoting an inflammatory microenvironment (IL-6, TNF-α), that collectively facilitate tumor migration and invasion.

DLL3 not only interacts with Notch receptors but it is also involved in cross-talk with other oncogenic signaling pathways that contribute to cancer progression (Figure 3). DLL3 activates the Wnt signaling pathway, which is required in various physiological processes [53]. DLL3 upregulates the ligands Wnt-1 and Wnt-4, as well as the downstream target genes of the Wnt pathway Axin-2 and Lef-1. The aberrant activation of the Wnt/β-catenin signaling pathway and downstream target genes contributes significantly to tumor initiation and progression [54]. The interaction between DLL3 and Wnt signaling leads to increased cancer cell survival and the promotion of a more aggressive phenotype. High DLL3 expression suppresses the AMPK signaling pathway, which is critical for the progression of colon adenocarcinoma. The inhibition of the Notch signaling pathway by DLL3 leads to the activation of the phosphoinositide-3-kinase/serine–threonine protein kinase B (PI3K/Akt) signaling pathway. DLL3 increases Akt phosphorylation in murine Lewis lung carcinoma cells, promoting their survival and reducing apoptosis [55]. Moreover, LIN28B and miR-518d-5p are upstream regulators of the DLL3-mediated proliferation and migration of SCLC [47]. High DLL3 expression in SCLC tumors induces impaired antitumor immunity characterized by the suppression of immune-related pathways and dendritic cell functions, as well as the reduced infiltration of T cells, macrophages, and dendritic cells relative to low-DLL3-expression cases (data cutoff date was 20 February 2023) [42]. High DLL3 expression induces a moderate to severe inflammatory infiltration and higher PD-L1 expression in SCLC compared to tumors expressing low DLL3 [56], suggesting a potential beneficial effect of immunotherapy in patients with positive DLL3 expression and active Notch 1 expression.

## 4. DLL3 as a Therapeutic Target in Cancer Treatment

**Figure 4:**
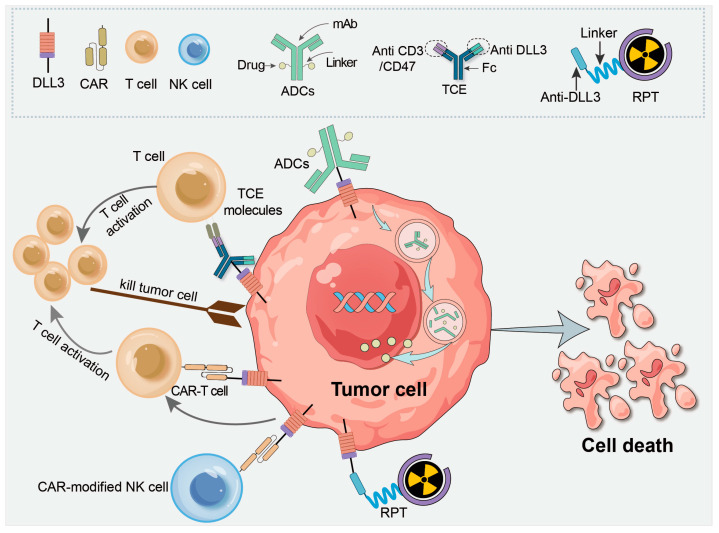
Mechanisms involved in DLL3-targeted cancer therapy. DLL3-targeted ADC: the anti-DLL3 antibody binds to tumor cells to stimulate endocytosis, thereby delivering the cytotoxic drug payload (e.g., the DNA alkylating agent in Rova-T) into the cell, leading to apoptosis. DLL3-targeted TCE molecules are bispecific antibodies that bind both CD3 on T cells and DLL3 on tumor cells, redirecting T cell cytotoxicity to specifically eliminate DLL3-expressing tumors. DLL3-targeted CAR-T/CAR-modified NK cells use cell-surface DLL3 as a target to induce immune cell cytotoxicity. DLL3-targeted RPT: the radiolabeled anti-DLL3 antibody delivers radiation, inducing lethal damage not only in tumor cells with high DLL3 expression but also in neighboring malignant cells (bystander effect). Abbreviations: ADC, antibody–drug conjugate; TCE, T cell engager molecule; CAR, chimeric antigen receptor; RPT: radiopharmaceutical therapy.

The results of pre-clinical and clinical trials indicate that the DLL3 located on cell membrane is a potential target for tumor treatment [8,21,57]. Various therapeutic approaches targeting DLL3 are being explored, including ADCs, precision immunotherapy, and radiopharmaceutical therapy (Figure 4). As regards ADCs, the anti-DLL3 antibody binds to tumor cells to stimulate endocytosis, thereby delivering the cytotoxic drug payload (e.g., the DNA alkylating agent in Rova-T) into the cell. In cellular immunotherapies involving CAR-T or CAR-NK cells, the objective is to use the cell-surface DLL3 as a target to induce immune cell cytotoxicity. In radiopharmaceutical therapy, the radiolabeled anti-DLL3 antibody delivers radiation, inducing lethal damage not only in tumor cells with high DLL3 expression but also in neighboring malignant cells (bystander effect). Table 1 lists the ongoing clinical trials targeting DLL3.

### 4.1. ADCs

ADCs represent the latest advance in precision oncology, as they target specific cell surface factors in various malignancies. Rovalpituzumab tesirine (Rova T) is the first-in-class ADC targeting DLL3 with promising results in preclinical models of SCLC and large-cell neuroendocrine carcinoma (NEC). It consists of a humanized DLL3-specific IgG1 monoclonal antibody linked to the DNA cross-linking agent pyrolobenzodiazepine through a protease-cleavable linker [8]. Rova T was used in a phase I study where it demonstrates durable responses and a safety profile, with a subsequent phase II trial in DLL3-expressing SCLC patients who showed a median overall survival (OS) of 5.6 months [9]. Rova T was also used in a phase I/II study (NCT02709889) involving patients with advanced solid tumors showing high DLL3 expression, and the results revealed that 0.3 mg/kg every 6 weeks for two cycles induce a manageable toxicity, with antitumor activity in patients with NEC [58]. However, the phase III trial in the TAHOE study (Rova T vs. topotecan) with second-line therapy for SCLC showed that the median OS was 6.3 months in the Rova T arm and 8.6 months in the topotecan arm [59]. The MERU study (Rova T vs. placebo) revealed that the median OS was 8.8 months in the Rova T arm and 9.9 months in the placebo arm [60]. Moreover, significant toxicity that induced pleural and pericardial effusions, photosensitivity reaction, and peripheral edema was observed [60]. Thus, further clinical investigations assessing the effect of Rova T in these trials were discontinued [61].

Nevertheless, Rova T enhanced the antitumor activity of anti-PD1 in a murine model of SCLC with DLL3 overexpression by activating dendritic cells and increasing Ccl5, IL-12, and ICAM [17]. Moreover, a new ADC targeting DLL3 was reported. Recently, Lin et al. developed a new anti-DLL3 ADC DB-1314, which induces potent, durable, and dose-dependent antitumor effects in vitro showing favorable pharmacokinetic and toxicokinetic profiles in rats and cynomolgus monkeys [62]. ZL-1310 and FZ-AD005 are new ADC generations, currently under clinical investigation (NCT06179069) to evaluate the safety, tolerability, and pharmacokinetics in SCLC and advanced solid tumors [63].

### 4.2. T Cell Engager (TCE) Molecules

TCEs have dual specificities, a characteristic that allows them to simultaneously bind to the CD3 complex on T cells and a target antigen on tumors. This dual binding effectively brings T cells in proximity to tumor cells, promoting T cell activation and proliferation and enhancing the immune response against tumor cells.

Tarlatamab (AMG757) is a first-in-class DLL3-targeted bispecific TCE, with dual affinity for DLL3 on tumor cells and CD3 on T cells [64,65]. Tarlatamab monotherapy used in preclinical studies shows the significant inhibition of tumor growth by promoting CD4+ and CD8+ T cell infiltration into patient-derived xenograft models of SCLC tumors and induces the release of the cytokines interferon IFN-γ, interleukin IL-6, IL-10, TNF-α, and IL-4. Tarlatamab possesses an excellent safety and efficacy profile in preclinical studies, making it a valid option in the clinical setting against SCLC expressing DLL3 [66]. Tarlatamab used in a phase I trial (NCT03319940) showed manageable safety, encouraging response durability, and a disease control rate of 51.4%. The median progression-free survival (PFS) and OS were 3.7 months and 13.2 months, respectively [67]. The phase II study (NCT05060016) evaluated patients with SCLC in whom two or more prior lines of treatment had failed and who received a dose of 10 mg every two weeks. The results demonstrated an objective response rate of 40% and a median PFS of 4.9 months. Treatment-related adverse events occurred in a low percentage (3%) of patients, causing the discontinuation of tarlatamab treatment only in this small percentage [68]. The results of the phase II trial accelerated the FDA approval of IMDELLTRA™ for the treatment of adult patients with extensive-stage (ES) SCLC with disease progression on or after platinum-based chemotherapy on 16 May 2024, and now IMDELLTRA is currently being evaluated in two phase III trials involving SCLC patients. The phase III DeLLphi-304 study (NCT05740566) is evaluating the effect of tarlatamab compared with that of standard care in subjects with relapsed SCLC after platinum-based first-line chemotherapy. The phase III DeLLphi-306 study (NCT06117774) is evaluating the effect of tarlatamab in subjects with limited-stage SCLC that did not progress following concomitant chemoradiation therapy. The third phase III study DeLLphi-305 (NCT06211036) plans to evaluate the effect of tarlatamab in combination with durvalumab vs. durvalumab alone in subjects with ES-SCLC following platinum, etoposide and durvalumab therapy, but patients have not yet been recruited according to the latest update posted on 10 May 2024. These results suggest that tarlatamab may represent a breakthrough in the treatment of patients with cancer showing high DLL3 expression, and further study is underway.

BI 764,532 is a bispecific TCE designed to target PD-1 and a tumor-associated antigen. It induces CD4+ and CD8+ T cells to attack DLL3-expressing cells, consequently inducing complete tumor regression in a human T cell-engrafted mouse model. Preclinical studies showed that BI 764,532 has similar pharmacokinetics to tarlatamab in non-human primates, with a half-life of 10 days [69]. Several clinical studies on BI 764,532 are currently underway, and they are listed in Table 1.

HPN328 is a trispecific T cell-activating protein involving a CD3 domain, a DLL3 domain, and an albumin domain for half-life extension, effectively inducing the immune response to attack and destroy cancer cells with high DLL3 expression. HPN328 dose-dependently upregulates CD25 and CD69 on T cells and induces the secretion of TNF-α and IFN-γ by T cells in the presence of DLL3-expressing tumor cells. Preclinical and nonclinical characterization suggests that HPN328 is highly effective and safe, thus being a potential new therapeutic candidate [28]. HPN328 shows linear pharmacokinetics in the administered dose range, with a serum half-life of 78 to 187 h [70]. It is currently being used in a phase I/II clinical trial (NCT04471727), with a dose escalation and expansion to assess the safety, tolerability, and pharmacokinetics of its use as monotherapy, as well as in combination with atezolizumab or ifinatamab deruxtecan (I-DXd) in patients with advanced high DLL3 expression tumors.

Other promising TCE molecules targeting DLL3, such as QLS31904, PT-217, RO7616789, and ZG006, are currently under evaluation in clinical trials, as listed in Table 1.

### 4.3. CAR-T Therapy

CAR-T cell therapy involves engineering T cells that recognize and attack tumor cells expressing specific antigens, which have been adapted to target DLL3 in tumor cells. CAR-T cell therapy has been highly successful against certain hematological malignancies; however, this has been much less the case in patients with solid tumors. DLL3-targeting CAR-T cells have been developed and shown promising results in preclinical studies by selectively killing DLL3-positive tumor cells [32]. Early-phase trials are ongoing to evaluate the safety and efficacy of this approach in patients with advanced SCLC.

AMG 119 is the first CAR-T cell therapy targeting DLL3; it is a genetically engineered T cell generated by transducing autologous T cells obtained using a self-inactivating lentiviral vector that encodes an anti-DLL3 target-binding domain, CD28 and 4-1BB co-stimulatory domains, and a CD3 domain [71]. The use of AMG 119 in preclinical studies revealed its specific cytotoxic activity against DLL3-expressing SCLC cells and antitumor activity in SCLC xenograft models. A phase I clinical trial (NCT03392064) using AMG 119 in patients with relapsed/refractory SCLC revealed its clinical safety and good tolerability at the tested doses, with encouraging results on cellular kinetics supporting the use of this CAR-T cell therapy in solid tumors [71]. Moreover, IL-18-secreting CAR-T cells increase the activation of both CAR-T cells and endogenous tumor-infiltrating lymphocytes, thus being a potentially promising new strategy against DLL3-expressing solid tumors [44]. Other in-human trials are currently underway, including a phase I trial (NCT05680922) studying LB2102 in ES-SCLC and LCNLC patients, a phase I trial studying SNC-115 in recurrent/refractory SCLC and neuroendocrine carcinoma (NCT06384482), and a phase I trial investigating BHP01, a DLL3-targeted α-PD-L1/4-1BB CAR-T (NCT06348797). An additional study involves ALLO-213, an allogeneic CAR-T developed by Allogene that targets DLL3 but has not yet been introduced into clinical trials.

### 4.4. CAR-Modified Natural Killer (NK) Cells

NK cells are critical members of the innate immunity lymphocytes and are involved in host defense against malignant cells. CAR-modified NK cells represent a promising immunotherapeutic approach to cancer treatment. At present, preclinical and clinical studies have shown that CAR-NK cell therapy exerts a significant antitumor effect, and it is safer than CAR-T cell therapy because CAR-NK cells have a shorter lifespan and limited cytokine secretion profile [72]. DLL3-specific NK-92 cells possess a NKG2D transmembrane domain and a co-stimulatory molecule 2B4-CD3 domain. Preclinical studies showed a potent and specific lytic activity and additional advantages, such as a donor-independent manufacturing process and “off-the-shelf” availability [73,74]. DLL3-CAR NK-92 cells induced tumor regression in an H446-derived pulmonary metastasis tumor model under a good safety threshold, indicating that these cells may represent a potential strategy in the treatment of SCLC [75]. A phase I trial involving DLL3-CAR-NK cells in patients with relapsed/refractory ES-SCLC (NCT05507593) recently started patient recruitment.

### 4.5. Radiopharmaceutical Therapy Targeting DLL3

Radiopharmaceutical therapy is increasingly recognized as a potentially safe and effective targeted treatment to cure several cancer types. Anti-DLL3 mAb SC16 radiolabeled with Lutetium-177 ([177Lu]Lu-DTPA-CHX-A”-SC16) markedly prolonged the survival of a patient-derived xenograft model, with a complete pathological response and mild and transient toxicity, thus being a potential approach for clinical translation [76,77]. Moreover, it may be useful to explore the well-documented immunomodulatory effects of radiotherapy on the tumor immune microenvironment, particularly on macrophages and myeloid-derived suppressor cells [16]; thus, it may be useful to explore synergistic effects with ICIs.

A DLL3-targeted imaging tracer has also been investigated. A phase I/II study on imaging using [(89)Zr]Zr-DFO-SC16.56 anti-DLL3 antibody revealed that DLL3 PET-CT in NEC is safe and feasible [78]. Targeting DLL3 for the diagnosis and treatment of tumors using [89Zr]-DFO-DLL3-scFv-integrated immunotherapy, AMG 757, and/or PET radiotracer in small-cell neuroendocrine prostate cancer may represent an effective approach [79].

## 5. Clinical Challenges in Targeting DLL3

### 5.1. Tumor Heterogeneity and Variability in DLL3 Expression

DLL3 expression is not uniform across tumor cells that constitute a tumor and among tumor cells of different tumors [80]. SCLC is divided into four subtypes: SCLC-A (ASCL1-driven), SCLC-N (NEUROD1-driven), SCLC-P (POU2F3-driven), and SCLC-I (inflamed/immune-rich). Among these, SCLC-A and SCLC-N show higher DLL3 expression, thus being correlated with an enhanced susceptibility to DLL3-targeted therapies, whereas SCLC-P and SCLC-I show lower DLL3 expression, potentially limiting the therapeutic efficacy [81,82]. In addition to SCLC, Spino et al. measured DLL3 expression in 34 different types of cancers by bioinformatics methods, revealing that DLL3 expression in isocitrate dehydrogenase (IDH)-mutant glioma, particularly in 1p/19q co-deleted subsets, is higher than that of IDH-wild-type glioblastoma [19]. This suggests that DLL3 may serve as a subtype-specific biomarker in gliomas, with implications for patient stratification. Moreover, DLL3 expression dynamically decreases and disappears as tumors evolve and progress due to genetic mutations, epigenetic changes (e.g., mutations in Notch pathway genes or promoter methylation), microsatellite instability, the tumor microenvironment (e.g., immune evasion or hypoxia-induced plasticity), and the stage of the disease [83,84,85]. DLL3 expression variability complicates patient selection for DLL3-targeted therapies and may reduce the effectiveness of these treatments in some individuals. Strategies to address these challenges include multiplexed biomarker testing and combination therapies targeting complementary pathways.

### 5.2. Resistance to DLL3-Targeted Therapies

Resistance to DLL3-targeted therapies represents another challenge. Tumors can adapt to DLL3-targeted treatments over time through various mechanisms, such as by downregulating DLL3 expression or upregulating compensatory signaling pathways that allow them to escape the effects of therapy. For example, DLL3 is expressed in the cytoplasm of normal hepatocytes but silenced in hepatocellular carcinoma cells because of DNA methylation and histone acetylation induced by the hepatitis B virus, which remodels the chromatin landscape to silence neuroendocrine genes [80,86]. Moreover, SCLC cells develop resistance to agents that target DLL3 by reverting to a non-DLL3-expressing phenotype or using alternative signaling pathways, such as the Notch, Wnt/β-catenin, MAPK/ERK, and PI3K/AKT pathways, which ensure tumor cell survival and proliferation in the absence of DLL3 [56]. Therefore, future research should focus on the investigation of the mechanisms regulating the resistance to DLL3-targeted therapies, to provide further new combination strategies (e.g., Notch/Wnt inhibitors or epigenetic modulators) that improve treatment response.

### 5.3. Adverse Effects and Barriers Associated with DLL3-Targeted Agents

DLL3-targeted therapies, including ADCs, are associated with several adverse effects despite the promising therapeutic benefits. Common adverse events reported in clinical trials include fatigue and nausea, revealing the cytotoxic nature of these agents [61]. Immunogenicity is also a potential concern, since it is influenced by the immune status of the patient, previous therapies, and the characteristics of the DLL3-targeted agent itself [87]. Some patients receiving DLL3-targeted therapies develop anti-drug antibodies, particularly with CAR-T cell therapies [88], meaning the development of a response against the infused CAR-T cells or the DLL3-targeted agents themselves, leading to a reduced efficacy and increased toxicity. The expression of the target antigen and an increase in the frequency of regulatory T cells impact the efficacy of TCEs [89,90]. Moreover, the presence of anti-drug antibodies induces an altered pharmacokinetics of the therapeutic agent, resulting in a reduced drug level in the blood stream, thus requiring dosage adjustments [91].

Effective patient classification is essential for the success of DLL3-targeted therapies, but several obstacles make this approach difficult [92,93]. First, the lack of standardized assays for the reliable detection of DLL3 expression leads to inconsistencies in identifying suitable candidates for treatment. Immunohistochemistry and RNA sequencing may give different results in terms of DLL3 expression, complicating the decision-making process for patient eligibility. Furthermore, comprehensive biomarker development is still in its infancy. The identification of biomarkers associated with treatment response is essential for maximizing the therapeutic potential of DLL3-targeted agents. The dynamic DLL3 expression in response to treatment or tumor evolution leads to discrepancies between the initial biopsy and subsequent assessment, making it difficult to determine the most suitable candidates for DLL3-targeted interventions. Therefore, it is of the utmost importance to understand the biological context of DLL3 expression, including its interaction with other pathways and tumor microenvironmental factors, for effective patient classification and appropriate selection.

## 6. Future Perspectives

The combination of DLL3-targeted therapies with other treatment modalities, such as immunotherapy or chemotherapy, is a promising area of exploration. For example, the combination of ADCs with immune checkpoint inhibitors like anti-PD-1 or anti-PD-L1 may enhance the therapeutic efficacy. This approach can help the immune system to recognize and attack cancer cells expressing DLL3 that may otherwise evade immune detection [94]. Rova T in combination with anti-PD-1 enhances the antitumor activity even at sub-effective doses in a murine SCLC tumor model [17]. The combination of checkpoint inhibitors with TCEs may mutually increase their antitumor effect. TCEs induce the upregulation of PD-1 and PD-L1 expression on immune and tumor cells, and the addition of PD-1 and PD-L1 inhibitors is associated with enhanced activity of both T cells and TCEs in tumors [17,95]. Thus, it may be possible to improve treatment outcomes and overcome resistance associated with monotherapies by targeting DLL3 while simultaneously boosting the immune response. Preclinical studies showed synergistic effects when DLL3-targeted therapies were used in combination with existing chemotherapeutic regimens [96], highlighting the potential improvement of the response rate and outcome (NCT04885998). Ongoing research and clinical trials are essential to understand the most effective combinations to optimize therapeutic strategies and obtain more personalized and effective cancer treatments.

The effect of DLL3-targeted therapies can be improved by advancements in drug delivery systems and innovative drug design. Next-generation ADCs are being developed to improve the specificity and potency of cytotoxic agents delivered to DLL3-expressing tumor cells [91]. These new ADCs not only use more potent chemotherapeutic agents but also new linker technologies to ensure stability in the blood stream before the delivery to the tumor site [97]. Moreover, innovations such as bispecific antibodies that target both DLL3 and other tumor-specific antigens increase the therapeutic effect by promoting dual targeting, potentially overcoming or reducing the limitations of tumor heterogeneity [98,99]. Another promising strategy involves the integration of nanoparticles or other new delivery systems that selectively deliver drugs to DLL3-expressing cells, minimizing the systemic exposure and associated toxicity [100,101]. The combination of liposomes and micelles is also a promising drug delivery system, which can be used to solubilize poorly water-soluble compounds, increasing the drug loading capacity and reducing off-target effects [102]. Innovative approaches including structure-based drug design allow the creation of small molecules that specifically inhibit DLL3 or its downstream signaling pathways. Moreover, emerging technologies like CRISPR/Cas9 can be used to create DLL3-specific therapies based on individual patient tumor profiles, tailoring the treatment to enhance efficacy. Ongoing research and collaboration among scientists, clinicians, and pharmaceutical industries are essential to translate these innovations into clinical practice, resulting in better outcomes for patients.

In conclusion, DLL3 expression is very low in normal adult tissues, while it is primarily observed in neuronal tissues (such as the spinal cord) and during embryonic development. Mature healthy tissues including those in the lung, liver, and kidney are characterized by a reduced or no expression of DLL3. This specificity increases the potential safety and effectiveness of therapies targeting DLL3. However, DLL3 is not only prominently highly expressed in SCLC but also in a variety of other malignancies, including neuroblastoma, pancreatic neuroendocrine tumors, glioma, ovarian cancer, and breast cancer. DLL3 overexpression in several tumors suggests its far-reaching potential as a therapeutic target. The ongoing investigation on the role and expression of DLL3 in different tumors is essential for identifying optimal treatment pathways and improving outcomes for patients with DLL3-expressing malignancies. This broad potential highlights the significance of DLL3 in future cancer research and therapeutic development.

## Figures and Tables

**Table 1:** Ongoing clinical trials targeting DLL3.

Agent	Targets	Conditions	Phase	Trial ID	Sponsor
**ADC**					
FZ-AD005	DLL3/Topoisomerase I Inhibitor	Advanced Solid Tumor SCLC, LCNC	I	NCT06424665	Zhangjiang Bio-Pharmaceutical
ZL1310	DLL3	SCLC	I	NCT06179069	Zai Lab (Shanghai)
**TCE**					
tarlatamab (AMG757)	DLL3/CD3	ES-SCLC	1b	NCT05361395 ^a^	Amgen Inc.
		LS-SCLC, SCLC	III	NCT06117774	
		ES-SCLC, SCLC	III	NCT06211036	
BI764532	DLL3/CD3	SCLC, Advanced NEC	I	NCT05879978 ^b^	Boehringer Ingelhelm
		SCLC, Other Neoplasms	I	NCT04429087	
		Relapsed/Refractory ES-SCLC, NEC	II	NCT05882058 ^c^	
		Advanced NEC	I	NCT06132113 ^d^	
		SCLC	1b	NCT05990738 ^e^	
		SCLC	I	NCT06077500 ^f^	
		Glioma	1b	NCT05916313	
		SCLC, NEC	I	NCT05963867 ^g^	
QLS31904	DLL3/CD3	Advanced Solid Tumor	I	NCT05461287	Qilu Pharmaceutical
PT-217	DLL3/CD47	Relapsed/Tefractory NEC	I/II	NCT05652686	Phanes Therapeutics
RO7616789	DLL3/CD3/CD137	SCLC, NEC	I	NCT05619744	Hoffmann-La Roche
HPN328	DLL3/CD3/albumin	Advanced Tumors	I/II	NCT00471727	Harpoon Therapeutics
ZG006	DLL3/DLL3/CD3	SCLC, NEC	I/II	NCT05978284	Suzhou Zelgen Bio-pharmaceuticals
**CAR-T**					
AMG119	DLL3/CD28/4-1BB/CD3	SCLC	I (suspended)	NCT03392064	Amgen
LB-2102	DLL3/DLL3	ES-SCLC, Lung LCNC	I	NCT05680922	Legend Biotech USA Inc
**CAR-NK**					
NK-92	DLL3	ES-SCLC	I	NCT05507593	Tianjin Cancer Hospital
**RPT**					
[177Lu]Lu-DTPA-CHX-A”-SC16	DLL3	NEC	I/II	NCT04199741	Memorial Sloan Kettering Cancer Center

Abbreviations: ADC, antibody-drug conjugate; TCE, T cell engager; CAR-T, chimeric antigen receptor T cell therapy; CAR-NK, chimeric antigen receptor nature killer cell therapy; RPT, radiopharmaceutical therapy; ES-SCLC, extensive-stage small-cell lung cancer; LS-SCLC, limited-stage small-cell lung cancer; LCNC, large-cell neuroendocrine carcinoma; NEC, neuroendocrine carcinoma; ^a^: combination with carboplatin, etoposide, and PD-L1 inhibitor; ^b^: combination with ezabenlimab; ^c^: DAREON™-5; ^d^: DAREON™-7, combined with standard of care (platinum and etoposide); ^e^: DAREON™-9, combined with topotecan; ^f^: DAREON™-8, combined with standard of care (platinium, etoposide, and anti-PD-L1); ^g^: PET imaging trial to investigate [89Zr]Zr-BI 764532 biodistribution and tumor uptake.

## Data Availability

Not applicable.
